# Influence of Early Postoperative Feeding in Gastrointestinal Anastomotic Fistula Formation and Healing Time in Rabbits

**DOI:** 10.1155/2018/8258096

**Published:** 2018-05-06

**Authors:** Ze Tang, Hongfei Cai, Youbin Cui

**Affiliations:** Department of Thoracic Surgery, The First Hospital of Jilin University, Jilin, China

## Abstract

**Objectives:**

To determine whether early postoperative feeding attenuates the inhibitory effects of intestinal anastomosis in rabbits.

**Methods:**

After undergoing gastrointestinal anastomosis, 48 rabbits were randomly divided into experimental and control groups. The rabbits in the experimental group were fed a liquid diet beginning 24 h postoperatively, while the control rabbits received only total parenteral nutrition after the operation. Exploratory laparotomies were performed on four rabbits in each group 3, 5, 7, 10, and 15 days postoperatively, and the healing rate of the anastomosis, anastomotic bursting pressure, anastomotic breaking strength, and hydroxyproline content at the anastomosis were determined.

**Results:**

The anastomoses healed in 91.6% (22/24) of the control group and 95.8% (23/24) of the experimental group. The anastomotic bursting pressure decreased remarkably in both groups 3 days postoperatively, reaching the lowest value. The anastomotic breaking strength did not differ between the two groups 3 days postoperatively, when both reached their lowest points, and both groups increased markedly and peaked 10 days postoperatively. The hydroxyproline content of the anastomosis was slightly lower in the experimental group 3 days postoperatively, although both groups peaked 7 days postoperatively.

**Conclusions:**

Early postoperative feeding does not increase the anastomosis healing time or rate of gastrointestinal anastomosis leakage.

## 1. Introduction

Gastrointestinal anastomosis is the most common gastrointestinal reconstruction surgery [1, 2]. A frequent complication after gastrointestinal anastomosis is gastrointestinal fistula formation, the incidence of which is 0–17.4% [3]. Mild cases can cause infection, electrolyte imbalance, and malnutrition, but severe cases may cause death. Anastomotic leakage is influenced by systemic factors, including diabetes, cirrhosis, and other chronic wasting diseases, which impair the body's repair capacity as well as its ability to fight infections and thus healing of the anastomosis. Many studies have shown that patients with a poor nutritional status are more prone to complications. A positive correlation between preoperative weight loss and anastomotic fistula was reported [4]. Insufficient mobility of the anastomosis, excessive resection, and excessive tension on the anastomotic site also compromise healing, as does an inadequate blood supply.

Wound healing is a process of dynamic equilibrium involving cells, their milieu, and the extracellular matrix [5, 6]. The cytokines secreted by platelets and inflammatory cells promote both the formation of new blood vessels and collagen synthesis, which in dynamic balance with collagen degradation determine the healing response [7]. Two important components of collagen are hydroxyproline and hydroxylysine. Hydroxyproline is synthesized under conditions of oxidative stress via the hydroxylation of proline and is involved in the cellular transport of collagen. The synthesis and transport of wound collagen can be understood by monitoring the hydroxyproline content of the wound [8].

Patients undergoing a gastrointestinal anastomosis are fed postoperatively via an indwelling gastric tube. Gastrointestinal decompression, fasting, and parenteral nutrition are also used to prevent postoperative nausea and vomiting. These methods provide sufficient time for the anastomosis to heal and for the gastrointestinal segment to regain its integrity. However, the feasibility of an alternative protocol, in which patients are given postoperative antiemetic drugs and fed a liquid diet in small quantities, with strict control of both the amount and frequency of intake to ensure that the anastomotic pressure remains within a safe range, has yet to be determined. A deficient nutritional status can be corrected quickly in a patient who, early after surgery, can tolerate oral enteral nutrition or the oral intake of homemade nutritious meals, perhaps in conjunction with parenteral nutrition support. This in turn accelerates recovery from the trauma of surgery [9].

The optimal method of controlling food intake after gastrointestinal anastomosis is unclear. The majority of surgeons consider that the later the normal eating is resumed, the more completely the gastrointestinal anastomosis will heal. However, after esophageal anastomosis in rabbits, better results were obtained with early rather than late feeding. In patients with gastrointestinal anastomosis, early feeding allows the early intestinal absorption of nutrients and thus improves tissue healing and reduces the incidence of postoperative gastric infection [10]. Early postoperative feeding is in line with the concept of rapid rehabilitation surgery, the aim of which is to reduce surgical stress and complications, accelerate recovery, shorten the length of hospitalization, lower nutritional costs, and improve recovery following physical and associated psychological trauma. The aim of this study was to examine the effect of early feeding on the healing of a gastrointestinal anastomosis in rabbit and thus, preliminarily, to elucidate the relationship between early feeding, gastrointestinal anastomotic fistula formation, and healing time after gastrointestinal surgery.

## 2. Materials and Methods

### 2.1. Experimental Animals

Forty-eight male and female rabbits (weight, 4–6 kg) used in this study were provided by the Basic Medical Laboratory Animal Laboratory of Jilin University School. The rabbits were randomly divided into experimental and control groups of 24 rabbits each. The test group was fed normal liquid food (ground rabbit chow diluted with warm water) 24 h postoperatively, with food intake strictly controlled. The initial feeding was 15 mL three times per day. Thereafter, single food was administered three times per day as follows: at 72 h postoperatively, 20 mL; at 5 days postoperatively, 25 mL; at 7 days postoperatively, 30 mL; at 10 days postoperatively, 35 mL; and at 15 days, 40 mL. The control group fasted after the operation, with water administered by intravenous infusion to maintain daily physiological requirements. The present study was approved by the Ethics Committee of The first hospital of Jilin University (Jilin, China) (number 2016-379).

### 2.2. Experimental Model

After fasting with water for 8 h before surgery, the rabbits were injected intramuscularly with anesthetic consisting of a mixture of ketamine (40 mg/kg) and droperidol (1.6 mg/kg). The abdomen of the rabbits was shaved, and disinfected, and the incision site treated with 5 mL of 2% lidocaine. A median abdominal incision was made, followed by subtotal gastrectomy and the creation of a gastric jejunum end-to-side full-thickness 1.2 cm anastomosis using 1-0 absorbable sutures and a stitch length of ~2 mm. After ensuring that there was no excess tension on the suture site and no active bleeding, the abdominal cavity was flushed with saline. Aseptic technique was maintained during the operation. The abdominal incision was sutured layer by layer. Four animals from each group were euthanized on postoperative days 3, 5, 7, 10, and 15. Tissue sections were prepared from day 5 samples and stained with hematoxylin and eosin (H&E) and Masson's trichrome staining. The following parameters were evaluated at each time point.

### 2.3. General Status

Postoperative food intake, weight changes, and wound healing were examined in the fasted control and fed experimental rabbits.

### 2.4. Healing Rate of the Anastomosis

The presence of pus and necrotic tissue around the anastomotic site and leakage of intestinal contents or other obvious signs of leakage were considered indicative of anastomotic fistula.

### 2.5. Rupture Pressure on the Anastomosis

The tissue 15 cm proximal and distal to the anastomotic site was excised and placed in Ringer's solution. One end of the segment was connected with a microinfusion pump and the other with a pressure tester. Methylene blue was injected at a rate of 8 mL/min using an infusion pump. The pressure causing overflow of the dye solution was defined as the anastomotic rupture pressure.

### 2.6. Immediate Pressure on the Anastomosis in the Experimental Group

The intestinal pressure caused by the maximal single food intake was imitated and the pressure on the anastomotic site recorded using the same method as described above.

### 2.7. Tensile Strength at the Anastomotic Site

Tissue 3 cm proximal and distal to the anastomotic site was excised and the remaining intestinal segment fixed to an ag-x plus Desktop 10 KN pneumatic chuck. Tension was applied at a tensile rate of 60 mm/min until rupture. The tensile strength was defined as the maximum load force causing interface rupture.

### 2.8. Morphology

The gastrointestinal wall tissues from the day 5 anastomoses were fixed in 10% neutral formaldehyde solution, paraffin-embedded, and processed for conventional tissue sectioning. The H&E-stained sections were examined by light microscopy for the amount of neovascularization and for fibroblast morphology, as indicators of anastomotic stoma healing. The Masson trichrome staining was examined by light microscopy for the amount the collagenous fiber. Image-Pro Plus Analysis Software helped us for calculating the collagenous fiber area ratio between the two groups. The result was automatic calculation by image analysis system.

### 2.9. Statistical Analysis

SPSS19.0 statistical software was used for the statistical analysis. Anastomotic rupture pressure and anastomotic tensile strength in the two groups of animals were compared and were expressed as the mean ± standard deviation. The mean values of the two groups were compared in a single factor analysis of variance (ANOVA). A* p* value < 0.05 was considered to indicate a statistically significant difference.

## 3. Results

### 3.1. General Status

After the operation, the two groups of animals were in good condition, and no deaths occurred. The mean postoperative weight of the rabbits in the experimental and control groups was 4.96 ± 0.42 kg and 5.04 ± 0.38 kg, respectively. On the 3rd postoperative day, the mean body weight of the experimental and control rabbits was 4.36 ± 0.28 kg and 4.41 ± 0.25 kg, respectively; postoperative day 5, 4.36 ± 0.28 kg and 4.41 ± 0.25, respectively; and postoperative day 10, 4.64 ± 0.28 kg and 4.77 ± 0.13 kg, respectively. None of the differences between the experimental and control groups were significant (*p* > 0.05). Infection of the incision did not occur in either group.

### 3.2. Healing Rate of the Anastomotic Site

Laparotomy was performed in the two groups of animals at the above-described postoperative time points. In general, the anastomosis and surrounding tissue and omentum differed in the degree of adhesion. Based on a definition of anastomotic fistula as the presence of pus and necrotic tissue or obvious leakage, none of the samples showed evidence of anastomotic fistula on day 3 postoperatively. A large number of peritoneal exudates were seen in the control group on the postoperative day 5, accompanied by an abscess that had formed around the anastomotic site and contamination with intestinal contents, confirming the occurrence of anastomotic fistula. On postoperative day 7, anastomotic fistula had occurred in both the control and the experimental group. The anastomotic healing rate was 91.6% (22/24) in the control group and 95.8% (23/24) in the experimental group. The difference in the healing rates of the two groups was not significant (*p* > 0.05).

### 3.3. Rupture Pressure of the Anastomotic Site

**Figure 1:**
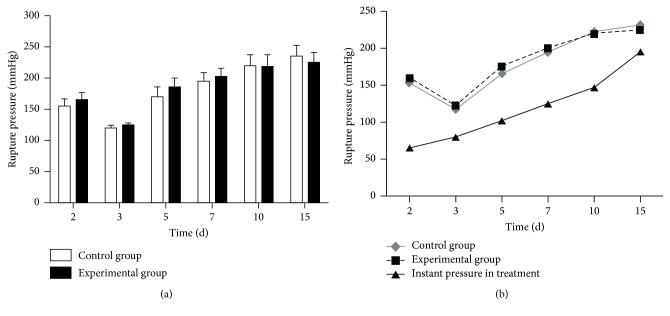
*Rupture pressure and instant pressure of the anastomotic site*. (a) On postoperative day 5, the rupture pressure increased significantly in the experimental and control groups, with a slightly lower pressure in the former. The peak rupture pressure of the control group was on postoperative day 7 and it decreased slightly on postoperative day 10. (b) With progressive healing of the anastomosis, the rupture pressure was higher than the immediate pressure.

There was no significant difference in the rupture pressures of the two groups on postoperative day 2, and the rupture pressure of both groups was significantly lower on day 3 than at any other time. On postoperative day 5, the rupture pressure increased significantly in the experimental and control groups, with a slightly lower pressure in the former. The peak rupture pressure of the control group was on postoperative day 7 and it decreased slightly on postoperative day 10, at which time the pressure in the experimental group reached a peak. On postoperative day 15, the pressure in the two groups was slightly lower than on postoperative day 10. However, there was no significant difference in the rupture pressure of the anastomosis between the two groups at any time point (*p* > 0.05) (Figure 1(a)).

### 3.4. Instant Pressure on the Anastomotic Site in the Experimental Group

As the mimicked food intake was gradually increased, the immediate pressure on the anastomotic site increased as well. With progressive healing of the anastomosis, the rupture pressure was higher than the immediate pressure. The values of the two pressures were the closest on postoperative days 3 and 15 (Figure 1(b)).

### 3.5. Tensile Strength

**Figure 2:**
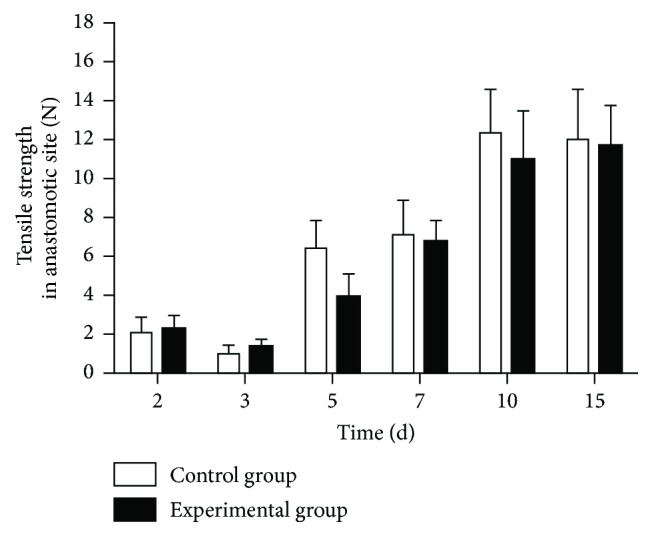
*Tensile strength on the anastomotic site*. On postoperative day 5, both groups had a significantly higher anastomotic tensile strength than on the previous days, with a slightly lower tensile strength in the experimental than in the control group.

There was no significant difference in the tensile strength of the anastomotic site between the two groups on postoperative day 2, although that of the control group was slightly lower. There was also no significant difference between the two groups on postoperative day 3. On postoperative day 5, both groups had a significantly higher anastomotic tensile strength than on the previous days, with a slightly lower tensile strength in the experimental than in the control group. On postoperative day 7, the anastomotic tensile strength of the experimental group was slightly but not significantly higher than that of the control group on postoperative day 5 and slightly lower than that of the control group on postoperative day 7. On postoperative day 10, the anastomotic tensile strength increased significantly in the two groups and reached a maximum in both. The anastomotic tensile strength of the experimental group was slightly lower than that of the control group. On postoperative day 15, the anastomotic tensile strength was slightly but not significantly lower than on day 10. There was no significant difference in the tensile strength of the anastomosis between the two groups at any of the time points (*p* > 0.05) (Figure 2).

### 3.6. Histology of the Postoperative Day 5 Anastomosis

**Figure 3:**
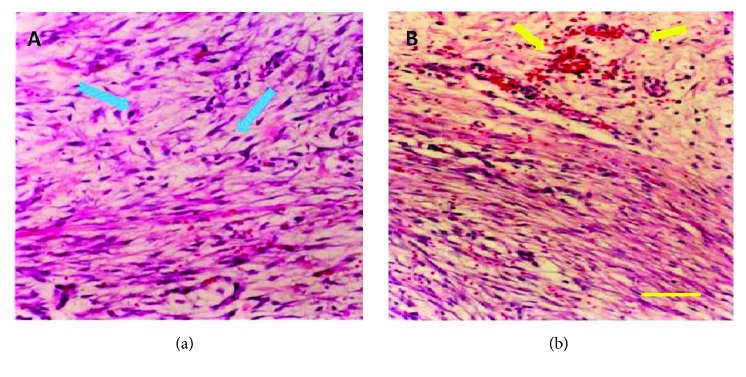
*Histology of the postoperative day 5 anastomosis (200x)*. Light microscopy of the H&E-stained postoperative day 5 sections showed rare neutrophils but large numbers of lymphocytes and monocytes in the anastomotic site tissues of both groups of animals. (a) Control group. Blue arrows showed cell necrosis. (b) Experimental group. Yellow arrows showed vascularization.

**Figure 4:**
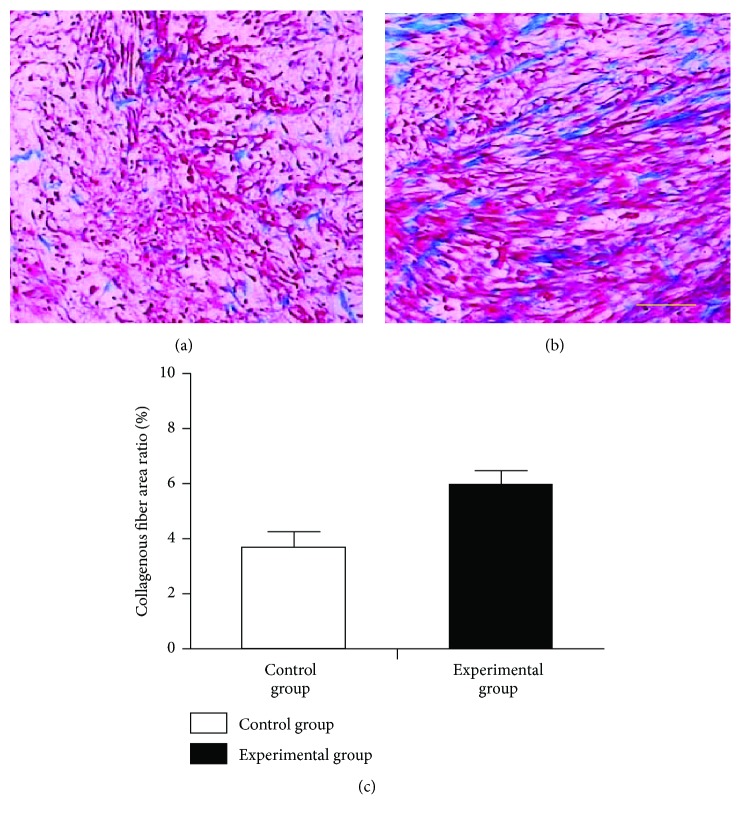
*Masson trichrome staining the postoperative day 5 anastomosis (200x)*. (a) Control group. (b) Experimental group. (c) The collagenous fiber area ratio is higher in experimental than the control group.

Light microscopy of the H&E-stained postoperative day 5 sections showed rare neutrophils but large numbers of lymphocytes and monocytes in the anastomotic site tissues of both groups of animals. Granulation tissue mainly composed of fibroblasts and new, ingrowing capillaries were seen on the wound surface (Figure 3). Vascularization could be obviously found in experimental group. Necrosis mucosa and submucosa tissue could be found in control group. Light microscopy of the Masson trichrome stained postoperative day 5 sections showed that different groups had different performance. The collagenous fiber area ratio is higher in experimental than the control group (Figure 4).

## 4. Discussion

Gastrointestinal anastomosis to reconstruct the integrity of the digestive tract is a highly invasive technique in general surgery. One of the more common complications after gastrointestinal anastomosis is gastrointestinal fistula, with an incidence of 0–17.4%. The optimal timing of postoperative feeding following gastrointestinal anastomosis is a matter of intense debate. Most surgeons believe that the postponement of eating protects the gastrointestinal anastomosis during the healing phase. However, previous studies in rabbits that underwent esophageal anastomosis demonstrated the advantages of early rather than late postoperative feeding, although this has not been confirmed in patients with gastrointestinal anastomosis. It can be argued that in gastric surgery patients the early intestinal absorption of nutrients will improve tissue healing and reduce the probability of postoperative infection [11, 12]. Moreover, early postoperative feeding is in line with the concept of rapid rehabilitation surgery to reduce surgical stress and complications, accelerate recovery, shorten the length of hospital stay, lower nutritional costs, and accelerate both physical and psychological healing in gastric surgery patients.

Further support for early feeding comes from studies showing that after the gastrointestinal procedure the small intestine usually reverts to normal function 4–8 h postoperatively, with normal function of the stomach and colon occurring somewhat later [13]. Thus, within 24 h postoperatively, eating can be tolerated and nutrients will be absorbed. In animal experiments, fasting resulted in a reduction in collagen content in the anastomotic scar tissue and therefore a reduced quality of healing, whereas early feeding prevented mucosal atrophy and increased collagen deposition and wound strength [14–16]. The results of other studies in animals and humans likewise suggested an association between nutrition via early feeding and improved healing of the anastomotic site [17–19].

Collagen synthesis and composition are important determinants of anastomosis healing. In our study, the anastomotic healing rate of the control and experimental groups was 91.6% and 95.8%, respectively. The tensile strength of the anastomotic stoma decreased significantly within 3 or 4 days after anastomosis. The tensile strength 48 h after gastroduodenal anastomosis was 64% lower, reflecting a decrease in the amount of collagen. This period is a critical phase of anastomotic healing, as there is a high risk of anastomotic leakage. By postoperative day 3 or 4, the accumulation of collagen had rapidly increased the tensile strength of the anastomosis.

In our experiment, the two groups did not differ significantly at any of the time points in terms of the rupture pressure or the tensile strength of the anastomosis. The strength of the anastomotic stoma in the early phase was mainly due to the tension of the sutures on the anastomosis. Our data are consistent with the results of previous studies showing that the anastomotic site was weakest 3 days after the operation and thus most vulnerable to fistula formation. Early in the tissue repair process, wound fibroblasts proliferate and synthesize abundant amounts of collagen, which together with granulation tissue capillaries play a key role in wound healing. Our results suggest that early postoperative eating does not affect the synthesis of collagen during healing of the anastomosis.

## 5. Conclusion

The results from this study indicate that with a gradual increase in single food intake, the pressure on the anastomotic site increases as well but can be maintained consistently lower than the burst pressure. Thus, early eating is safe and feasible, as long as it is strictly controlled with respect to maximum food intake. The number of daily feedings must be increased appropriately to ensure that the anastomotic pressure remains lower than the burst pressure, so as to avoid anastomotic fistula formation. As the anastomosis heals, food intake can be gradually increased. This regimen will reduce surgical stress and complications, accelerate patient rehabilitation, shorten the hospital stay, lower nutritional costs, and accelerate both the physical and the psychological healing of the patient.

## Data Availability

The data in the manuscript are original and available.
